# Rapid Review of Real-World Cost-Effectiveness Analyses of Cancer Interventions in Canada

**DOI:** 10.3390/curroncol29100574

**Published:** 2022-09-30

**Authors:** Andrea M. Guggenbickler, Heather K. Barr, Jeffrey S. Hoch, Carolyn S. Dewa

**Affiliations:** 1 Graduate Group in Public Health Sciences, Department of Public Health Sciences, University of California, Davis, CA 95616, USA; 2 Division of Health Policy and Management, Department of Public Health Sciences, University of California, Davis, CA 95616, USA; 3 Center for Healthcare Policy and Research, University of California, Davis, CA 95820, USA; 4 Department of Psychiatry and Behavioral Sciences, University of California, Davis, CA 95817, USA

**Keywords:** cost-effectiveness, cancer interventions, real-world interventions, cancer, economic evaluation, healthcare

## Abstract

Cost-effectiveness analysis (CE Analysis) provides evidence about the incremental gains in patient outcomes costs from new treatments and interventions in cancer care. The utilization of “real-world” data allows these analyses to better reflect differences in costs and effects for actual patient populations with comorbidities and a range of ages as opposed to randomized controlled trials, which use a restricted population. This rapid review was done through PubMed and Google Scholar in July 2022. Relevant articles were summarized and data extracted to summarize changes in costs (in 2022 CAD) and effectiveness in cancer care once funded by the Canadian government payer system. We conducted statistical analyses to examine the differences between means and medians of costs, effects, and incremental cost effectiveness ratios (ICERs). Twenty-two studies were selected for review. Of those, the majority performed a CE Analysis on cancer drugs. Real-world cancer drug studies had significantly higher costs and effects than non-drug therapies. Studies that utilized a model to project longer time-horizons saw significantly smaller ICER values for the treatments they examined. Further, differences in drug costs increased over time. This review highlights the importance of performing real-world CE Analysis on cancer treatments to better understand their costs and impacts on a general patient population.

## 1. Introduction

Economic evaluation produces important results that inform healthcare funding decisions in Canada. Not only do economic evaluations like cost-effectiveness analysis (CE Analysis) provide evidence about the costs and effects of new treatments and interventions for healthcare payers, they also provide patients and providers with comprehensive evidence in order to inform their decisions. This is especially important when considering expensive, new cancer treatments and interventions in Canada. 

For example, in Canada’s public health care system, cancer drugs are reviewed by Heath Canada for safety and efficacy. The pan-Canadian Oncology Drug Review (pCODR), through its expert review committee, makes reimbursement recommendations for oncology pharmaceuticals to the participating federal, provincial, and territorial publicly funded drug programs. The pCODR also makes recommendations related to the identification, evaluation, and promotion of responsible drug prescribing and use in Canada [1]. The expert review committee uses a deliberative framework to ensure the consistency and transparency of its cancer drug review process, and this framework includes cost-effectiveness as key component [1]. 

The trial-based evidence that informs the economic models used to estimate cost-effectiveness often comes from brief “controlled” trials with rigid study protocols, involving special oncologists and some of their unique patients. Use of these data is the cornerstone of evidence-based recommendations before a cancer drug is funded. After a drug is funded, however, data exist about the real-world costs and outcomes, since healthcare payers generally keep track of their beneficiaries’ costs and mortality status. Real-world CE Analysis provides information about a new drugs’ extra cost (ΔC) and extra effect (ΔE) after it has been funded and used by real clinicians and their potentially less healthy, less young, and less adherent patients [2]. In this paper, we review the Canadian literature on the real-world CE Analysis of recent cancer treatments and interventions. We summarize our findings and provide suggestions for next steps. 

### Background

Cost-effectiveness analysis (CE Analysis) studying a new treatment or intervention’s extra cost (ΔC) and extra effect (ΔE) can be conducted by analyzing a dataset or by synthesizing a body of evidence using a decision analytic model. Before a drug is funded, often phase III randomized controlled trials (RCTs) provide this type of data. Besides the concerns related to generalizability of the clinicians and patients, RCTs may have challenges related to timing. For example, Haslam et al. (2022) found that the median duration of treatment in studies initially testing a drug was 6.0 months (range: 2.2–12.7 months), whereas the median duration of treatment when the same drug was used as a comparator was 4.9 months (range: 1.7–12.0 months) [3]. Of course, treatment duration outside of an RCT may differ. In addition, Del Paggio et al. (2021) found median follow-up has decreased over three time periods from 47 months (1995–2004) to 37 months (2005–2009) to 25 months (2010–2020) [4]. They conclude that contemporary oncology RCTs now largely measure surrogate end points, such as disease-free and progression-free survival (PFS) and are almost exclusively funded by the pharmaceutical industry. Del Paggio et al. (2021) [4] also note that, “while there are some contexts in which PFS is an appropriate end point, this is the exception and not the rule; in most contexts, PFS is not a valid surrogate” for quality of life or length of life [5,6,7,8].

Using data from studies that could have benefited from a longer duration means either having to use surrogate outcomes or having missing survival time data. In these situations, researchers resort to economic modeling to connect secondary outcomes (e.g., life years) to primary outcomes (e.g., quality adjusted life years or QALYs), or to extend the analysis time horizon from something convenient to something useful. The first part of any debate about the cost-effectiveness of a cancer treatment involves the estimates of ΔC and/or ΔE. A CE Analysis model synthesizing the available evidence is all there is to go on before a cancer drug or intervention is funded. However, after a drug is funded, data exists on how much more expensive (or cheaper) a new treatment is and how much more (or less) effective it is. In general, payers know what they are paying and whether patients are alive. Therefore, it is possible with data routinely collected by a Provincial Cancer Agency or a Ministry of Health (MOH) to estimate ΔC and ΔE. There are both familiar (e.g., missing outcome data) and new challenges (e.g., no randomization) that accompany the use of real-world data, however. Methods for statistical cost-effectiveness analysis as well as economic models provide ways to address many of these challenges. 

In the past, there has not been a formal process introducing to decision makers and healthcare funders the results of real-world CE Analysis; nevertheless, researchers have contributed examples of real-world CE Analysis to the scientific literature. However, recently, Canada has created a real-world evidence Working Group to develop guidance on real-world studies for the purpose of health technology assessment in Canada [9]. Therefore, the insights that come from a review of real-world CE Analysis in oncology are useful for informing new plans for optimizing access (via public funding) to both current and future treatments and interventions. While there are many reasons to believe that a single cancer treatment’s ΔC, ΔE, and their ratio called the incremental cost-effectiveness ratio (ICER) may differ based on evidence available before versus after funding, it is important to learn from a summary of the published findings from Canadian real-world CE Analyses that have the potential to inform the role of future of real-world evidence initiatives in Canadian oncology.

## 2. Methods

Our team conducted a rapid review of existing literature following the Preferred Reporting Items for Systematic Reviews and Meta-Analyses (PRISMA) guidelines [10]. Ethics review was not needed, as this research was conducted using solely publicly available information sources and published articles.

### 2.1. Search Strategy 

This review focused on the utilization of “real-world” data for CE Analysis of cancer treatments in Canada. “Real-world” in the context of this review was defined as using data from a cohort, observational study or using individual-level person data from administrative or clinical databases. If evaluating pharmaceuticals, data collection had to occur after public funding. After initial widespread searches, a narrower search strategy was developed with the goal of finding studies fulfilling all of the search criteria. Thus, terms of “Canada,” “Cancer,” “Cost-effectiveness,” and “Real-world” (and variations) were used in the search strategy.

Example Search: “Canada AND (cost-effectiveness OR costs) AND cancer AND (real-world OR observational).”

### 2.2. Information Sources

The research team conducted a primary review in July of 2022 using PubMed, and Google Scholar to potentially identify new sources. Google Scholar yielded no new results. Given the nature of the review and the focus on Canada, all studies selected were based in Canada with a focus on cancer treatments or interventions (e.g., treatment, screening, etc.). Studies needed to include CE Analysis information, and contain information on ΔC and ΔE. ICERs were either obtained directly from the articles, or hand calculated based upon ΔC and ΔE if possible (e.g., ICER = ΔC/ΔE). All studies needed to include analysis on a “real-world” population, as per the definition above. There was no date restriction on the search. 

### 2.3. Screening Process 

Each article included in this review was identified through a systematic review process including (1) title screening, (2) abstract screening, (3) full text review, and (4) full text extraction. At each phase, two reviewers (AMG and HKB) independently screened each article, with JSH serving as arbiter. Despite having an arbiter to make the final decision, disagreements were discussed among all reviewers for a consensus. Covidence software [11] was used to ensure anonymous reviewing, ease of conflict resolution, and to calculate interrater reliability. Study inclusion and exclusion criteria were applied to each phase of review and can be found in Table 1. We calculated a Kappa statistic to measure agreement between the reviewers using the equation: [(% agreement) − (% agreement based on chance)]/(1 − % agreement based on chance).

### 2.4. Conversions and Calculations 

If a paper presented a range for any ΔC, ΔE, or ICER, the values were averaged to create a single number for data visualization. To summarize results for comparison, we adjusted all ΔCs reported in Canadian dollars (CAD) to 2022 Canadian dollars using the Bank of Canada inflation calculator [12]. One study, reported in United States Dollars (USD), was first converted to 2022 value [13], and then to CAD [14]. Once we standardized ΔCs to 2022 CAD, we calculated the ICER as ΔC/ΔE. 

### 2.5. Estimates by Study Type 

As an exploratory analysis of whether estimates differed by study type, we choose four study type classifications as a matter of sample size and convenience. These study types were defined as binary variables indicating if the research was (i) studying a drug; (ii) using a model; (iii) using the QALY as an outcome; or iv) was recent (i.e., after 2017). We compared means and medians for the estimates of ΔC, ΔE and the ICER. Monetary values were converted to 2022 Canadian dollars as described above. To test differences in the means, we conducted *t*-test assuming unequal variances between groups. To test differences in the medians we used a non-parametric test on the equality of medians with chi-squared test statistics computed with a continuity correction. The t-test and median testing were done in Stata using the commands t-test and median, respectively [15]. 

### 2.6. Estimates of Extra Cost and Extra Effect in Real World Studies of Drugs 

To illustrate estimates for studies of drugs, we plotted ΔC and ΔE on a cost-effectiveness plane. We picked an arbitrary willingness-to-pay (WTP) value of λ = CAD 100,000 to provide context about the cost-effectiveness.

## 3. Results

### 3.1. Article Inclusion and Exclusion Results

**Figure 1:**
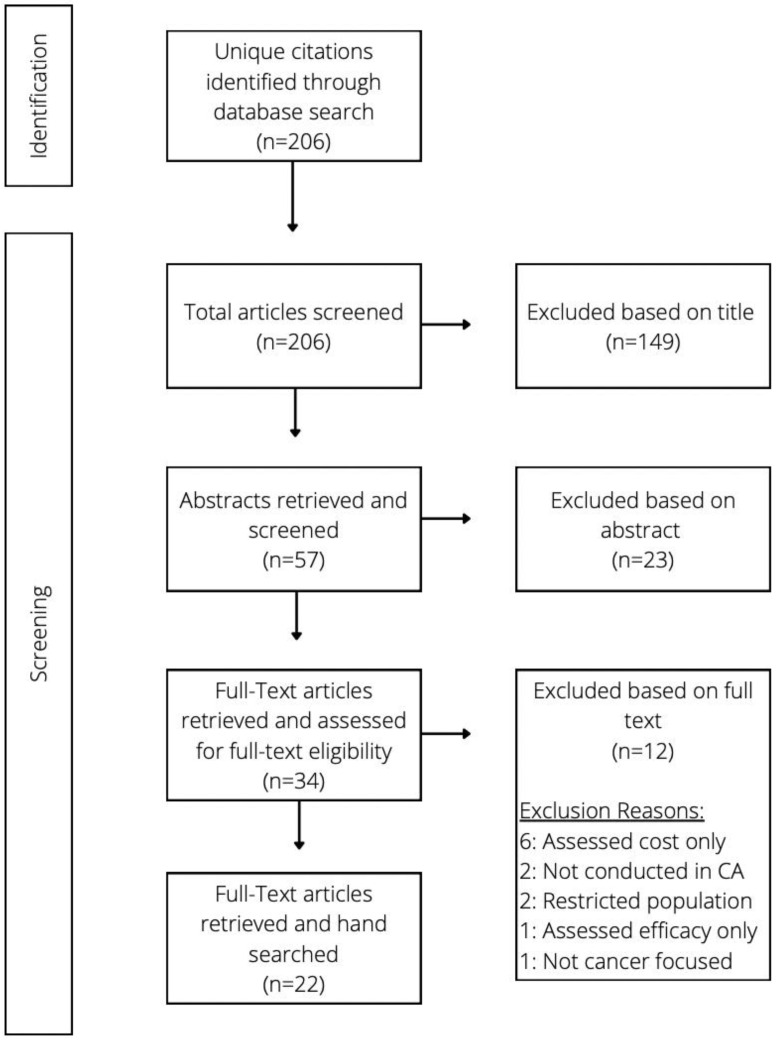
Flowchart of literature search and selection.

The search of the literature in the PubMed database resulted in the identification of 206 unique articles. After conducting the title review, 149 articles were excluded, leaving 57 articles for abstract review. After conducting the abstract review, 23 articles were excluded, leaving 34 to be included in the full-text review. Reasons for exclusion in the full text review portion of the process were: (1) Did not include cost-effectiveness analysis (e.g., only focused on cost or effect), (2) Incorrect setting (e.g., not conducted in Canada), (3) Used a restricted population (e.g., utilized data from a randomized controlled trial), or (4) Did not focus on a cancer intervention. After the full text review, 22 articles remained for data extraction (Figure 1). Overall interrater reliability based on title review, abstract review, and full text review was calculated as 0.87, strong agreement. 

### 3.2. Overview of the Studies

The majority of Canadian studies (59%) examining real-world cost effectiveness focused on drugs (Table 2). A smaller portion (41%) of studies focused on non-drug interventions such as screening, surgical interventions, and genetic sequencing. From the 22 studies, we were able to extract 29 (ΔC, ΔE) pairs and compute 27 ICERs. ICERs were not computed for new drugs that were less effective than usual care, as best practice is not to report negative ICERs [16].

**Table 2:** Description of studies.

Citation	Intervention(s)	Sample Size (n)	Study Population	Study Design, Study Perspective, Time Horizon	Measures of Effectiveness
Arciero et al., 2022 [17]. JNCI Cancer Spectr.	Comparing cost-effectiveness of using gemcitabine and nab-paclitaxel (Gem-nab) and FOLFIRINOX for patients with advanced pancreatic cancer.	n = 1988; Gem-nab = 928; FOLFIRINOX = 1060	Aged ≥ 18 years that were prescribed Gem-nab, irinotecan, or oxaliplatin for advanced pancreatic cancer between 17 April 2015–31 March 2019. Mean age (SD) of groups ranged from 61.9(8.8)–69.2(9.0).	Study Design: Dataset; Study Perspective: Healthcare System; Time Horizon: 5 years	QALYs, LYs
Cressman et al., 2021 [18]. CMAJ Open	Comparing digital breast tomosynthesis (DBT) plus digital mammography (DM) in high-risk cancer screening to standard care (DM alone).	n = 112,249	Participants aged 40–74 years who participated in breast cancer screening from the BC Cancer Breast Screening Program and the BC Cancer Registry for all new screening participants, mean age (SD): 49.3 (40–74) years, with an initial, “index,” screening exam received between 1 January 2012, and 31 December 2017.	Study Design: Model; Study Perspective: Healthcare System; Time Horizon: Lifetime	QALYs
Cressman et al., 2017 [19]. J. Thorac. Oncol. Off. Publ. Int. Assoc. Study Lung Cancer.	Comparing high-risk lung cancer screening to standard care. The base case scenario drew a comparison of LDCT- based screening in the HR-NLST (intervention) with the high-risk CXR (HR-CXR) screened arm (comparator) of the NLST, using the assumption that CXR is similar to standard care for early lung cancer detection.	n = 49,775	Data from the National Lung Cancer Screening Trial (NLST). Participants were separated into high (>2% at 6 years) and low risk (<2% at 6 years) groups. The outcomes data for NLST participants were grouped according to risk (high or low) and screening intervention (LDCT or chest radiography [CXR]).	Study Design: Model; Study Perspective: Public Payer; Time Horizon: 30 years (lifetime horizon)	
Cromwell et al., 2011 [20]. Lung Cancer Amst. Neth.	Comparing third-line erlotinib protocol to the next-best alternative of Best Supportive Care (BSC) in BCCA patients.	n = 147; erlotinib = 78, supportive care = 69	BCCA cases with a diagnosis of stage IIIB/IV advanced NSCLC (including adenocarcinoma, NSC carcinoma, squamous cell and large cell carcinomas, bronchio- alveolar carcinoma, and lung carcinomas not otherwise specified). Median age (SD) erlotinib: 65(39–88); best supportive care: 64(45–77).	Study Design: Dataset; Study Perspective: Healthcare System; Time Horizon: N/A	
Cromwell et al., 2011 [21]. J. Thorac. Oncol. Ogg. Publ. Int. Assoc. Study Lung Cancer.	Second-line erlotinib treatment and treatment with docetaxel among patients with non-small cell lung cancer.	n = 201; erlotinib = 133, docetaxel = 68	Eligible patients were patients treated at the BCCA with a diagnosis of stage IIIb/IV advanced NSCLC who received second-line treatment (including adenocarcinoma, NSC carcinoma, squamous cell and large cell carcinomas, bronchioloalveolar carcinoma, and lung carcinomas not otherwise specified). Median age receiving Erlotinib: 65(39–88). Median age (SD) receiving Docetaxel: 64(45–77).	Study Design: Dataset; Study Perspective: Provincial Healthcare System; Time Horizon: N/A	LYs
Dai et al., 2022 [22]. JAMA Oncol.	Treatment with pertuzumab, trastuzumab, and chemotherapy after public funding compared with treatment with trastuzumab and chemotherapy before funding in patients with metastatic breast cancer.	n = 1158	Patients who received first-line treatments for metastatic breast cancer from 1 January 2008, to 31 March 2018, were identified. Mean age (SD) of patients: 58(12.97 years).	Study Design: Dataset; Study Perspective: Public Payer; Time Horizon: N/A	QALYs, LYs
Gilbert et al., 2020 [23]. J. Comp. Eff. Res.	Treating recurrent high-grade serious ovarian cancer with cytotoxic chemotherapy and after relapse.	n = 66	Mean age (SD) was 60.6 (8.6) years, and 48% of the women were Caucasian. At diagnosis, 68% had stage IIIC, and 25% had stage IV ovarian cancer.	Study Design: Dataset; Study Perspective: Healthcare System; Time Horizon: N/A	LYs
Hannouf et al., 2012 [24]. BMC Cancer	Utilizing a 21-gene recurrence score assay to inform treatment decisions for women with early-stage breast cancer.	n = 498; Pre-menopausal: 109, Post-menopausal: 389	Pre- and post-menopausal women, average age (≈64 years old) living in Manitoba and have been diagnosed with ER+/PR + LN- ESBC (stage I/II) breast cancer between January 2000–December 2002.	Study Design: Model; Study Perspective: Healthcare System; Time Horizon: Lifetime horizon	QALYs, LYs
Hedden et al., 2012 [25]. Eur. J. Cancer Oxf.	Comparing (1) mCRC therapy for all patients in the post-bev era compared to usual mCRC therapy in the pre-bev era; and (2) mCRC therapy for patients diagnosed under age 70 and who received doublet chemotherapy (i.e., those eligible for bevacizumab) in the post-bev era compared to usual mCRC therapy for the same sub-group of patients in the pre-bev era.	n = 943	All patients with newly diagnosed mCRC referred to BCCA were included; pre-bev cohort: 2003–2004, post-bev cohort: 2006. Mean age at diagnosis: 65.	Study Design: Model; Study Perspective: Healthcare Payer; Time Horizon: 12 years	QALYs, LYs
Hedden et al., 2012 [26]. The Oncologist	Adjuvant trastuzumab for operable, HER-2/neu-positive early breast cancer with standard of care treatments in the adjuvant and metastatic settings.	n = 1000	50-year-old women with early HER- 2/neu-positive breast cancer, who had successfully completed a surgical resection of disease. Patients entered the model in the postsurgical with trastuzumab or postsurgical without trastuzumab states, depending on the presence or absence of pre-existing low left ventricular ejection fraction (LVEF).	Study Design: Model; Study Perspective: Healthcare System; Time Horizon: 28 years	QALYs, LYs
Imran et al., 2019 [27]. Eur. Thyroid J.	Primary versus tertiary care among patients with low-risk differentiated thyroid cancer.	n = 317; Tertiary care = 224, Primary care = 93	Patients diagnosed with low risk differentiated Thyroid Cancer diagnosed between 1 January 2006 and 31 December 2011. Mean age at diagnosis, tertiary: 47.7; mean age at diagnosis, primary care: 46.0.	Study Design: Dataset; Study Perspective: Healthcare System, Patient Perspective; Time Horizon: N/A	Rate of Recurrence
Johnston et al. 2010 [28]. ParmacoeconimcsOutcomes Res.	Cyclophosphamide, doxorubicin, vincristine, and predisone (CHOP) chemotherapy versus CHOP-R (CHOP with the addition of rituximab) in the treatment of large B-cell lymphoma.	n = 785	Patients with diffuse large B-cell lymphoma. Sample included 266 HIV-negative adults (age > 15 years) initiating treatment with CHOP between September 1997 and June 2000 and 519 HIV-negative adults initiating treatment with CHOP-R between March 2003 and June 2007.	Study Design: Model; Study Perspective: Healthcare Payer; Time Horizon: 15 years	QALYs, LYs, Disease-free LYs
Khor et al., 2014 [29]. BMC Cancer	Treating diffuse-large-B-cell lymphoma with rituximab plus cyclophpsphamide, doxorubicin, vincristine, and predisone (CHOP) (RCHOP) as opposed to only CHOP.	n = 4021; RCHOP = 2825, CHOP = 1196	All ages diagnosed with diffuse-large-B-cell-lymphoma were included in data collection from the date of rituximab approval. Data were collected on patients from 1997–2009. Mean group ages ranged from 56.7–65.5; included patient populations ≥80 years.	Study Design: Dataset; Study Perspective: Healthcare System; Time Horizon: 5 years	LYs
Mittmann et al., 2018 [30]. J. Clin. Oncol Off. J. Am. Soc. Clin. Oncol.	Use of 21-gene assay Oncotype Dx test or standard of care for chemotherapy prescription.	n = 1000	Patients with hormone receptor–positive breast cancer who received endocrine therapy. Median age (SD) at registration: 58 (50–65 years).	Study Design: Database; Study Perspective: Healthcare System; Time Horizon: N/A	Chemotherapy Use Rates
Nazha et al., 2018 [31]. Curr Oncol Tor Ont.	First line treatment in a real world setting for patients with metastatic renal cell carcinoma using sunitinib versus pazopanib.	n = 475; sunitinib = 395, pazopanib = 80	Patients diagnosed with clear cell metastatic renal cell carcinoma after 1 January 2011. Median age (SD): 63 (56–70).	Study Design: Database; Study Perspective: Healthcare Payer; Time Horizon: N/A	LYs
Nazha et al., 2018 [32]. Drug Investig.	Suntinib versus pazopanib in patients with metastatic renal cell carcinoma.	n = 475	mRCC patients treated with targeted therapy (sunitinib or pazopanib) in first-line with confirmed clear cell histology. Mean age: 64.	Study Design: Model; Study Perspective: Healthcare System; Time Horizon: 5 years	QALYs, LYs
Parackal et al. 2020 [33]. Can Urol. Assoc. J. J. Assoc. Urol. Can.	Use of robot-assisted radical prostatectomy (RARP) for prostate cancer or open radical prostatectomy (ORP).	n = 14,396; RARP v ORP developing BCR = 8259, Fail point of BCR = 1485, UI after RP = 2510, ED after RP = 2142	Men with localized prostate cancer, stages I and II. Mean ages 50–69 years old.	Study Design: Model; Study Perspective: Public Payer; Time Horizon: 10 years	QALYs
Pataky et al., 2021 [34]. MDM Policy Pract.	Use of bevacizumab-treated patients versus those treated before bevacizumab funding and contemporaneous controls (receiving chemotherapy without bevacizumab) among patients with metastatic colorectal cancer.	n = 16,250; Ontario = 12,112, Saskatchewan = 1161, British Columbia = 2977	Metastatic Colorectal Cancer patients (greater than or equal to 18 years old at diagnosis) with a registry-confirmed diagnosis of colorectal cancer (ICD-O-3 codes C18-C20), who initiated irinotecan-based chemotherapy between 1 January 2000, and 31 March 2015 (BC and Ontario), or 1 January 2003, and 31 December 2015 (Saskatchewan). Mean age between 63 and 64 years.	Study Design: Dataset; Study Perspective: Public Payer; Time Horizon: 5 years	QALYs, LYs
Raymakers et al., 2020 [35]. BMC Cancer	Use of brentuximab vedotin (BREN + AVD) or ABVD (doxorubicin, bleomycin, vinblastine, and dacarbazine) as frontline therapy in patients with advanced Hodgkin’s lymphoma.	n = 1519	Patients diagnosed with advanced stage Hodgkins’s Lymphoma between 2000–2016, ≥18 years old, not pregnant, not HIV positive.	Study Design: Model; Study Perspective: Healthcare System; Time Horizon: 15-year	QALYs
Tesch et al., 2022 [36]. Cancer.	Chemotherapy prescription and RS-guided treatment costs post-TAILORx.	n = 2066; pre-funding: 644, post-funding = 739, post-TAILORx cohort = 683	HR-positive, HER2-negative, node-negative patients with breast cancer defined by diagnosis: before RS funding (cohort 1 [C1]: January 2013–December 2013), after introduction of public RS funding (cohort 2 [C2]: July 2015–June 2016), and after TAILORx results (cohort 3 [C3]: July 2018–June 2019). Patients aged 18–80 years with stage I–III breast cancer. Cohort 1 median age (SD): 62(23–80), Cohort 2: 62(21–80), Cohort 3 61(28–80).	Study Design: Dataset; Study Perspective: Healthcare Payer; Time Horizon: N/A	Chemotherapy Use Rates
Thein et al., 2017 [37]. Cancer Med.	Use of transarterial chemoembolization (TACE) + radiofrequency ablation (RFA) versus RFA monotherapy versus no treatment.	n = 2222	All eligible Hepatocellular carcinoma (HCC) cases aged 18 years and older in Ontario diagnosed between 1 January 2002 and 31 December 2010.	Study Design: Dataset; Study Perspective: Healthcare Payer; Time Horizon: N/A	QALYs, LYs
Weymann et al., 2021 [38]. J Community Genet.	Using genomic sequencing to tailor cancer care.	n = 460; usual care = 230, POG intervention = 230	Adults with varying metastatic, uncurable cancer types. Mean age (SD), usual care: 56.5(11.4); POG patients: 56.2(12.8).	Study Design: Dataset; Study Perspective: Healthcare Payer; Time Horizon: 1 year	LYs

#### 3.2.1. Description of Study Populations and Participants

In general, most of the studies selected for review used datasets with sample sizes of 1000–2000 patients, but some had upwards of 10,000 observations. For example, Pataky et al. analyzed 16,250 observations [34], with 12,112 from an Ontario cohort, 1161 from a Saskatchewan cohort, and 2977 from a British Columbia cohort. Cressman et al. boasts a cohort of 112,249 for their analysis of a screening program [18]. Given the real-world nature of these studies, the study populations more accurately reflect actual patient populations than clinical trial populations. For example, Khor et al. [29] included patients over the age of 80 with diffuse-large-B-cell-lymphoma. Not only is the age of these patients remarkable, but this patient population is also more likely to have confounding comorbidities; something not common in RCTs with “ideal” study populations. Similarly, other studies included in the review often cited a mean age of over 60 years of age, with common median and mean ages between 60 and 64 [17,20,21,23,24,25,29,31,32,33,34,36]. 

#### 3.2.2. Diagnoses

Based on our inclusion criteria, studies focused on interventions for different types and stages of cancer. The most common type of cancer studied was breast cancer; one article focused on screening, while the others focused on various stages and types, for example, early stage, metastatic, or hormone receptor-positive breast cancer [18,22,24,26,30,36]. Other studies focused on metastatic colorectal cancer (mCC) [25,34], metastatic renal cell carcinoma (mCRC) [31,32], diffuse large B-cell lymphoma [28,29], bronchioalveolar carcinoma (BCCA) stage IIB/IV [20,21], as well as advanced pancreatic cancer [17], lung cancer [19], ovarian cancer stage IIC/IV [23], low-risk thyroid cancer [27], prostate cancer [33], advanced stage Hodgkin’s lymphoma [35], hepatocellular carcinoma [37], and metastatic, incurable cancer types [38]. 

#### 3.2.3. Study Designs

A small majority (59%) of studies utilized purely administrative or retrospective data to inform their calculations and studies. The rest (41%) used these administrative and retrospective data to inform models for cancer interventions. Commonly used datasets came from Cancer Care Ontario’s New Drug Funding Program (NDFP) database [39], the BC Cancer Agency Information System (CAIS) database [40], the pan-Canadian Early Detection Lung Cancer Study (PanCan) [41], Ontario Cancer Registry [42], and the Canadian Kidney Cancer Information System (CKCis) database [43].

#### 3.2.4. Cost Perspectives

All studies cited governmental healthcare payers as the cost perspective. Given the nature the Canada Health Act (CHA) which aims to ensure that all eligible Canadians have access to prepaid insured health services, it is reasonable for studies to utilize a governmental payer perspective, especially if researchers seek to inform government funding decisions [44].

#### 3.2.5. Measures of Effectiveness

Measures of effectiveness were based on four main outcomes: (1) Quality adjusted life years (QALYs), (2) Life years (LYs), (3) Chemotherapy use rates, and (4) Recurrence rates. A majority (nine) of studies measured effectiveness in both QALYs and LYs [17,22,24,25,26,28,32,34,37]. Six of the studies measured effectiveness in only LYs, [20,21,23,29,31,38] while four measured in only QALYs [18,19,33,35]. Two utilized chemotherapy use rates as their measure of effectiveness [30,36], and one utilized rate of recurrence [27].

### 3.3. Results of the Studies

Table 3 shares the results of data extraction from the 22 articles. Table 3 shows currency in the year presented in the original papers. All of the studies included were published between 2012 [28] and 2022 [17,22,36]. While the majority of papers (59%) looked at the real-world cost effectiveness of drugs on cancer, 41% focused on other aspects of cancer care: 7 on screening methods [18,19,23,24,30,36,38], 1 on preventing recurrence [27], and 1 on assistive surgery [33].

**Table 3:** Results of studies.

Author(s)	Intervention(s)	ΔE	ΔC	ICER/ICUR(s)	Currency	Uncertainty
Arciero et al., 2022 [17]. JNCI Cancer Spectr.	Comparing cost-effectiveness of using gemcitabine and nab-paclitaxel (Gem-nab) and FOLFIRINOX for patients with advanced pancreatic cancer.	Gem-nab v FOLFIRINOX: −0.25 LY, −0.21 QALY	Gem-nab v FOLFIRINOX: CAD 2366	-	2019 CAD	SA, CEAC, Scatter plot
Cressman et al., 2021 [18]. CMAJ Open	Comparing digital breast tomosynthesis (DBT) plus digital mammography (DM) in high-risk cancer screening to standard care (DM alone).	DBT + DM v DM: 0.027 QALY	DBT + DM v DM: CAD 470	DBT + DM v DM: CAD 17,149/QALY	2019 CAD	SA
Cressman et al., 2017 [19]. J. Thorac. Oncol. Off. Publ. Int. Assoc. Study Lung Cancer.	Comparing high-risk lung cancer screening to standard care. The base case scenario drew a comparison of LDCT- based screening in the HR-NLST (intervention) with the high-risk CXR (HR-CXR) screened arm (comparator) of the NLST, using the assumption that CXR is similar to standard care for early lung cancer detection.	High-risk screening v Standard care: 0.032 QALY *Authors’ Assumptions*	High-risk screening v Standard care: CAD 668 *Authors’ Assumptions*	High-risk screening v Standard care: CAD 20,724/QALY *Authors’ Assumptions*	2015 CAD	SA
Cromwell et al., 2011 [20]. Lung Cancer Amst. Neth.	Comparing third-line erlotinib protocol to the next-best alternative of Best Supportive Care (BSC) in BCCA patients.	Third-line erlotinib v best practice: 0.25 LY	Third-line erlotinib v best practice: CAD 11,102	Third-line erlotinib v best practice: CAD 36,838/LY	2009 CAD	SA, Scatter plot
Cromwell et al., 2011 [21]. J. Thorac. Oncol. Ogg. Publ. Int. Assoc. Study Lung Cancer.	Second-line erlotinib treatment and treatment with docetaxel among patients with non-small cell lung cancer.	Second-line erlotinib v Docetaxel: 0.0027 LY	Second-line erlotinib v Docetaxel: CAD 2891	Second-line erlotinib v Docetaxel: CAD 1,055,215/LY	2009 CAD	SA, CEAC, Scatter plot
Dai et al., 2022 [22]. JAMA Oncol.	Treatment with pertuzumab, trastuzumab, and chemotherapy after public funding compared with treatment with trastuzumab and chemotherapy before funding in patients with metastatic breast cancer.	Pertuzumab addition v Previous care: 0.61 LY, 0.44 QALY	Pertuzumab addition v Previous care: CAD 192,139	Pertuzumab addition v Previous care: CAD 316,203/LY, CAD 436,679/QALY	2018 CAD	CEAC, Scatter plot
Gilbert et al., 2020 [23]. J. Comp. Eff. Res.	Treating recurrent high-grade serious ovarian cancer with cytotoxic chemotherapy and after relapse.	One line v Two lines: 1.17 LY Two lines v Three lines: −7.9 months	One line v Two lines: CAD 72,374; Two lines v Three lines: CAD 97,243	One line v Two lines: CAD 62,040/LY	2016 CAD	Not stated
Hannouf et al., 2012 [24]. BMC Cancer	Utilizing a 21-gene recurrence score assay to inform treatment decisions for women with early-stage breast cancer.	21-gene assay v Standard clinical practice. Pre-menopausal: 0.05 QALY; Post-menopausal: 0.062 QALY	21-gene assay v Standard clinical practice. Pre-menopausal: -CAD 50; Post-menopausal: CAD 3700	21-gene assay v Standard clinical practice. CAD 60,000/QALY for post-menopausal women	2010 CAD	SA, CEAC, Scatter plot
Hedden et al., 2012 [25]. Eur. J. Cancer Oxf.	Comparing (1) mCRC therapy for all patients in the post-bev era compared to usual mCRC therapy in the pre-bev era; and (2) mCRC therapy for patients diagnosed under age 70 and who received doublet chemotherapy (i.e., those eligible for bevacizumab) in the post-bev era compared to usual mCRC therapy for the same sub-group of patients in the pre-bev era.	Pre- v post- bevacizumab: 0.06 QALY, 0.325 LY	Pre- v post- bevacizumab: CAD 3791	Pre- v post- bevacizumab: CAD 43,058/QALY, CAD 10,764/LY	2009 CAD	SA, CEAC, Scatter plot
Hedden et al., 2012 [26]. The Oncologist	Adjuvant trastuzumab for operable, HER-2/neu-positive early breast cancer compared with standard of care treatments in the adjuvant and metastatic settings.	Adjuvant trastuzumab v Standard care: 1.38 QALY, 1.17 LY	Adjuvant trastuzumab v Standard care: CAD 18,133	Adjuvant trastuzumab v Standard care: CAD 13,095/QALY, CAD 15,492/LY	2009 CAD	SA, CEAC, Scatter plot
Imran et al., 2019 [27]. Eur. Thyroid J.	Primary versus tertiary care among patients with low-risk differentiated thyroid cancer.	Tertiary care v Primary care: 0.4% less recurrence *Authors’ Assumptions*	Tertiary care v Primary care: CAD 46.11	Tertiary care v Primary care: CAD 11,528/recurrence avoided	2017 CAD	Not stated
Johnston et al. 2010 [28]. ParmacoeconimcsOutcomes Res.	Cyclophosphamide, doxorubicin, vincristine, and predisone (CHOP) chemotherapy versus CHOP-R (CHOP with the addition of rituximab) in the treatment of large B-cell lymphoma.	CHOP-R v CHOP: Younger individuals: 0.6 LY, 0.5 QALY, 0.8 disease-free LY; Older individuals: 1.7 LY, 1.4 QALY, 1.9 disease-free LY	CHOP-R v CHOP. Younger individuals: CAD 9572; Older individuals: CAD 8194	CHOP-R v CHOP. Younger individuals: CAD 15,953/LY, CAD 19,144/QALY, CAD 11,965/disease-free LY; Older individuals: CAD 4820/LY, CAD 5853/QALY, CAD 4313/disease-free LY	2006 CAD	SA, Scatter plot
Khor et al., 2014 [29]. BMC Cancer	Treating diffuse-large-B-cell lymphoma with rituximab plus cyclophpsphamide, doxorubicin, vincristine, and predisone (CHOP) (RCHOP) as opposed to only CHOP.	RCHOP v CHOP: 0.27 LY	RCHOP v CHOP: CAD 16,298	RCHOP v CHOP: CAD 61,984/LY	2009 CAD	CEAC, Scatter plot
Mittmann et al., 2018 [30]. J. Clin. Oncol Off. J. Am. Soc. Clin. Oncol.	Use of 21-gene assay Oncotype Dx test or standard of care for chemotherapy prescription.	Addition of assay: 23% less chemotherapy rX	Addition of assay: CAD 3000	Addition of assay: CAD 13,043/chemo treatment avoided	2014 CAD	Not stated
Nazha et al., 2018 [31]. Curr Oncol Tor Ont.	First line treatment in a real world setting for patients with metastatic renal cell carcinoma using sunitinib versus pazopanib.	Sunitinib v Pazopanib: 0.43 LY	Sunitinib v Pazopanib: CAD 24,232	Sunitinib v Pazopanib: CAD 56,353/LY	2017 CAD	Not stated
Nazha et al., 2018 [32]. Drug Investig.	Suntinib versus pazopanib in patients with metastatic renal cell carcinoma.	Sunitinib v Pazopanib: 1.21 LY; 0.54 QALY	Sunitinib v Pazopanib: CAD 36,303	Sunitinib v Pazopanib: CAD 30,002/LY; CAD 67,227/QALY	2017 CAD	SA, Scatter plot
Parackal et al. 2020 [33]. Can Urol. Assoc. J. J. Assoc. Urol. Can.	Use of robot-assisted radical prostatectomy (RARP) for prostate cancer or open radical prostatectomy (ORP).	RARP v ORP: 0.0662 QALY	RARP v ORP: CAD 1701	RARP v ORP: CAD 25,704/QALY	2019 CAD	SA, CEAC
Pataky et al., 2021 [34]. MDM Policy Pract.	Use of bevacizumab-treated patients versus those treated before bevacizumab funding and contemporaneous controls (receiving chemotherapy without bevacizumab) among patients with metastatic colorectal cancer.	Bevacizumab v Past standard care: 0.4–0.83 LY	Bevacizumab v Past standard care: CAD 31,200–66,600	Bevacizumab v Past standard care: CAD 78,000–CAD 84,000/LY	2019 CAD	SA
Raymakers et al., 2020 [35]. BMC Cancer	Use of brentuximab vedotin (BREN + AVD) or ABVD (doxorubicin, bleomycin, vinblastine, and dacarbazine) as frontline therapy in patients with advanced Hodgkin’s lymphoma.	BREN + AVD v ABVD: 0.46 QALY	BREN + AVD v ABVD: CAD 192,336	BREN + AVD v ABVD: CAD 418,122/QALY	2018 CAD	SA, CEAC, Scatter plot
Tesch et al., 2022 [36]. Cancer.	Chemotherapy prescription and RS-guided treatment costs post-TAILORx.	Use of RS after funding v before: 19% decrease in chemotherapy use; Use of TAILORx v RS: 23% decrease in chemotherapy use	Use of TAILORx v RS: CAD 145,612	Use of TAILORx c RS: CAD 633,096/chemo treatment avoided	2021 CAD	SA
Thein et al., 2017 [37]. Cancer Med.	Use of transarterial chemoembolization (TACE) + radiofrequency ablation (RFA) versus RFA monotherapy versus no treatment.	TACE + RFA v No treatment: 0.93 QALY; RFA v No treatment: 0.88 QALY	TACE + RFA v No treatment: CAD 2304; RFA v No treatment: CAD 13,697	TACE + RFA v No treatment: CAD 2465/QALY; RFA v No treatment CAD 15,553/QALY	2013 USD	SA, CEAC, Scatter plot
Weymann et al., 2021 [38]. J Community Genet.	Using genomic sequencing to tailor cancer care.	Genomic sequencing v Usual care: 0.0025 LY	Genomic sequencing v Usual care: CAD 5203	Genomic sequencing v Usual care: CAD 2,081,200/LY	2015 CAD	Not stated

Table acronyms: LY: Life Year, QALY: Quality Adjusted Life Year, SA: Sensitivity analysis, CEAC: Cost effectiveness acceptability curve, CAD: Canadian dollars.

#### 3.3.1. Difference in Effectiveness

The range of LYs gained ranged between –0.66 [23] all the way up to 1.7 [28] among the studies that focused on drug treatments, and 0.06 [25] through 1.4 [28] for QALYs. Gilbert et al. [23] and Arciero et al. [17], reported the only negative values for ΔE, indicating that the intervention was less effective than the standard treatment. The range of ΔEs was smaller for studies that did not focus on drug therapies. ΔEs involving QALYs for non-drug therapies ranged from 0.027 [18] to 0.0662 [33]. Only one study investigated LYs for non-drug therapies which resulted in a ΔE of 0.0025 [38] (Table 3).

#### 3.3.2. Costs

Once all costs were converted to 2022 CAD, studies with the outcomes of LYs and QALYs were broken into the same categories as above: drug therapies and other cancer investments. Of these, drug therapies had, on average, higher ΔCs compared to the other cancer care investments (CAD 51,489 vs. 2,872, respectively). From 2010, the incremental cost effectiveness ratio (ICER) for drug therapies has increased. The average ICER before 2018 was CAD 126,449.90 and 338,009.02. While the difference was not statistically significant (*p* = 0.36) the estimated change in ICER value for the two time periods was CAD 211,559.12 (Table 4). For drug therapies, the average ICER values were CAD 134,841/QALY and 187,549/LY. Non-drug approaches to cancer care had a lower average ICER at CAD 37,993/QALY. Only one study examined LYs gained for non-drug cancer care. Weymann et al. [38] reported low observed survival gain (0.0025 LYs or a little less than one more day) with an ICER of CAD 2,501,696/LY.

#### 3.3.3. Estimates by Study Type

Table 4 shows that estimates of ΔC, ΔE, and the ICER did not differ statistically by study type, for the most part. The estimates of the means and the medians do appear to the eye to differ in certain cases; however, because of small sample size, it is likely there is not enough power to detect a statistically significant difference. For example, as mentioned above, the means for ICERs before and after 2018 seem differ by over CAD 200,000; however, the *p*-value for their difference is greater than 5%. This is seen as well with the ICER using QALYs versus using LYs; there is a difference of over CAD 200,000 but an insignificant *p*-value (*p* = 0.29). Studies of drugs had a significantly higher ΔCs (*p* < 0.05) and ΔEs *p* < 0.001) compared to non-drug studies pharmaceuticals. While the ICERs for drug studies vs. non-drug studies differed by over CAD 300,000, this was not statistically significant (*p* = 0.49). The median ICERs produced by modeling studies was significantly smaller than that produced by person-level data; however, there was no difference in the mean, likely as a result of skewed data and small sample size. Lastly, average ΔC after 2017 was significantly higher (*p* < 0.05) than in the earlier period.

#### 3.3.4. Estimates of Extra Cost and Extra Effect in Real World Studies of Drugs

**Figure 2:**
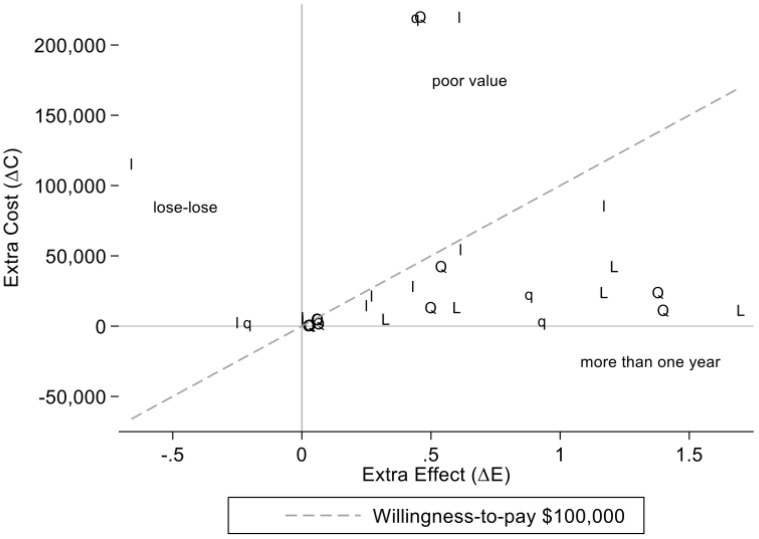
Estimates of Extra Costs and Extra Effects in real world studies of drugs. Q or L indicate QALYs and Life Years estimates of ΔE from a Model. In contrast, q or l indicate qalys and life years estimates of ΔE from a person-level analysis (non-Model). Data are only from studies of drugs.

Figure 2 shows estimates of ΔC and ΔE plotted with “Q” or “L” for models using QALYs or Life Years as the outcome. Studies that analyzed a dataset (non-modeling studies), have “q” or “l” to indicate the use of qalys or life years as the outcome. Most of the estimates appear below the dashed willingness-to-pay line with slope CAD 100,000. 

Studies with results to the left of the vertical line at 0, indicate “lose-lose” situations where an option may actually be less effective but more costly. There are three estimates like this in Figure 2 (two using life years and one using qalys). Toward the top of Figure 2, there are three studies in a “poor value” neighborhood. These studies provide evidence of drugs with little extra effect with extra costs in excess of CAD 200,000. Six of the 24 points in Figure 2 have extra effect estimates greater than one year. A majority of these (5 of 6) are estimates from models.

## 4. Discussion

Our study reviewed Canadian research exploring ‘value’ in the real-world, and summarized CE Analyses from Canadian cancer studies to identify post-funding ΔCs, ΔEs, ICERs and outcome measures. Systematically reviewing these CE Analysis studies provides additional insight; it allows us to identify trends and overall findings at an aggregate level. Our findings show it is possible for researchers to utilize “real-world” databases (e.g., administrative data) to compare incremental cost, incremental effect, and incremental cost effectiveness ratios for different cancers. However, our results also indicate that there is a dearth of CE Analyses for select cancers. This suggests that real-world CE Analysis may not be a feasible option for all cancer types and treatments.

While funding decisions are often informed by CE Analysis results and RCT data, this evidence is from a restricted population that is selected based on strict criteria that make RCT participants different from common cancer patients. Not only does this mean that those who would not quality for trials may not have access to treatments that might have been effective for them, but it also leaves physicians and patients with little concrete information about how to proceed with treatment decisions. The studies in our review conduct CE Analysis using a “real-world” population using administrative databases and medical records. This allows readers to understand what the extra gains and costs are when treating common real-world patients with novel treatments or interventions. Oftentimes, these studies acknowledge the difference in ΔCs (generally higher) [20,21], ΔEs (generally lower) [22,29] leading to higher ICERs when comparing their real-world results to those from RCTs [21]. 

Many of the papers we reviewed provide evidence of cost-effectiveness of cancer drugs in the real world as demonstrated by the marker position under the WTP dashed line in Figure 2. However, there are some instances where treatments show poor cost-effectiveness in the real world. For example, Arciero et al. [17] found that first-line gemcitabine plus nab-paclitaxel (Gem-Nab) was dominated by fluorouracil, folinic acid, irinotecan, oxaliplatin (FOLFIRINOX) in patients with advanced pancreatic cancer, as it was less effective and more costly. There are no RCTs comparing Gem-Nab to FOLFIRINOX. Likewise, Gilbert et al. [23] studying repeated cytotoxic chemotherapy treatments for recurrent high-grade serous cancer (HGSC) of the ovaries found that after the third relapse of HGSC, cytotoxic chemotherapy did not prolong survival but was associated with substantially increased healthcare costs. These findings from Arciero et al. [17] and Gilbert et al. [23] provide valuable feedback about real world use of treatments that are both more costly for payers and less effective for patients. They also help demonstrate why both pre- and post- funding CE Analyses are valuable—such studies can help calibrate decision making processes with real-world evidence. 

When real world data are combined in a decision analytic model, it is possible to extend past conventional study time horizons. Figure 2 shows this benefit commonly associated with models as a majority of the largest extra effect estimates (i.e., ΔEs) come from model-based analyses. The largest gain from treatment was 1.7 additional life years (indicated with an “L” on the far right of Figure 2). Johnston et al. [28] created this estimate over a 15-year time horizon using a patient-level simulation model for diffuse large B-cell lymphoma (DLBCL) patients initiating treatment with cyclophosphamide, doxorubicin, vincristine, and predisone (CHOP) chemotherapy versus the addition of rituximab to CHOP (CHOP-R). Khor et al. [29] also studied CHOP-R vs. CHOP in DLBCL patients using a cost-effectiveness dataset. They estimated a life expectancy increase of 3.2 months over five years; this corresponds to 0.27 life years (3.2 months/12 months). Thus, models provide more time to see potential gains from treatment. In fact, even within their own data, Khor et al. [29] were able to show how a longer analysis time horizon could boost extra gains; RCHOP was associated with a mean absolute survival gain of approximately 1.3 months (95% CI 0.7–2.3) at three years but it increased to 3.2 months (95% CI 1.6–4.7) at five years. Adding two more years of analysis to the study time horizon increased the ΔE estimate by 1.9 months, more than 146%. The cost of a longer time horizon is often more uncertainty. One can see this in the larger 95% CI for Khor et al.’s five-year ΔE estimate compared to the three-year 95% CI [29]. 

After adjusting for inflation, studies focusing on cancer drugs had a significantly higher ΔC after 2017. This larger ΔC may reflect rising drug costs, as all costs in Table 4 are presented in 2022 Canadian dollars. While the difference in the ICER was not statistically significant, the estimates were drastically higher post-2017 whereas there was no difference in the incremental effects of the drugs. One approach to improving ICER values in real-world settings is to lower drug costs. The lower numerator (ΔC) in the ICER calculation results in a more favorable ICER when considering patients treatment. In real-world analysis, we observed increasing costs and the use of modeling to have a significant impact on the overall value of cancer care. These findings must be interpreted with caution as hypothesis generating. However, utilizing the results of this review as a baseline, it is possible to, with future research, continue to refine clinical trial results to reflect “real-world” evidence. As our review has shown that real-world CE Analysis is possible, future post-funding analyses are indicated in order to validate findings, gain experience, and build community [45].

### Strengths and Limitations

There are several potential limitations to this review. First, given the large number of publication databases, it is possible that our search did not include every relevant article. However, given that we found 22 articles to be included after full-text review, it is very likely that we gathered enough source material to convey accurate impressions about the state of the field. Second, currently published real-world CE Analyses may not be representative of results from unpublished real-world studies. It is possible that published research represents the results from studies that are feasible to do (e.g., fast acting cancers). This bias is akin to survivorship bias, as only the studies that can survive the research and publication processes survive to make it into print. Lastly, due to the nature of our review, our findings are intended to inform and promote future research and research directions, rather than directly inform policy or treatment decisions about any particular drug. 

Major strengths of our review include a systematic search of PubMed for Canadian economic evaluations of cancer treatments or interventions using real-world data. For our research, two reviewers were used and a third one served as arbiter. This study summarizing Canadian results in this way is one of the first of its kind. We found a variety of published examples from real-world analyses of current cancer treatments that were (1) cost-effective (below the WTP line); (2) not cost-effective (above the WTP line); substantially more effective (with ΔE > 1), and even less effective (with ΔE < 0).

## 5. Conclusions

Economic evaluations like cost-effectiveness analysis (CE Analysis) are key to describing value and efficiency. Our findings illustrate the importance of analyzing value pre- and post- funding, considering study design, use of modeling, and time horizon. The results of randomized controlled trials (RCTs) have long been used in funding decisions for cancer care and interventions, but RCTs do not generally use a representative patient population. By looking instead at using real-world observational data on the same treatments through a CE Analysis lens, we see how these interventions will impact the average patient and the public healthcare payer. This type of research allows for the examination of real-world costs and effectiveness of new cancer treatments and interventions, accounting for patient diversity, long-term effects, and generalizability to Canada’s cancer patient population. Utilizing real-world data allows for true, large-scale CE Analysis on patient populations who are the actual consumers of cancer interventions and who have not been screened out for the sake of drug approval and funding decisions. 

## Figures and Tables

**Table 1:** Study Inclusion and Exclusion criteria.

**Study Inclusion Criteria**
Study takes place in Canada (province or territory). Study must focus on cancer and cancer interventions (treatment or prevention). Study or article conducts or models a cost-effective analysis on cancer treatments/prevention. Study or article must focus on a “real-world” population. Articles must be in English.
**Study Exclusion Criteria**
Study does not take place in a Canada province or territory. Study does not focus on cancer and/or cancer treatment/prevention. Study or article does not conduct a cost-effectiveness analysis, or does not do such on the provision of cancer treatments/prevention. Study or article does not focus on a “real-world” population. Study or article discusses only an RCT design with restricted populations. Article is not available in English. Article is a review (systematic or otherwise).

**Table 4:** Estimates by study type.

Study Type	Extra Cost (ΔC) In 2022 Canadian Dollars (CAD)		Extra Effect (ΔE), as Either Life Years or QALYs		Incremental Cost-Effectiveness Ratio (ICER) (CAD)	
	Mean	Median	Mean	Median	Mean	Median
Drug						
Yes (n = 24)	50,396.70	22,283.62	0.58	0.52	149,963.21	47,085.67
No (n = 5)	2872.24	1908.17	0.04	0.03	530,733.24	28,824.32
*p*-value for difference	0.003 **	0.06	<0.001 ***	0.06	0.49	0.93
Model						
Yes (n = 15)	28,081.67	11,441.67	0.64	0.5	63,058.95	25,092.81
No (n = 14)	57,332.64	22,283.62	0.32	0.35	417,247.72	76,724.32
*p*-value for difference	0.25	0.2	0.13	0.35	0.14	0.04 *
QALYs as outcome						
Yes (n = 15)	41,002.70	8238.84	0.47	0.45	105,812.63	26,731.66
No (n = 14)	43,322.95	21,650.43	0.5	0.43	326,949.47	62,577.42
*p*-value for difference	0.93	0.35	0.9	0.85	0.29	0.56
After 2017						
Yes (n = 14)	74,524.06	42,566.94	0.32	0.44	338,009.02	76,044.77
No (n = 15)	12,036.35	11,441.67	0.64	0.5	126,449.90	25,092.81
*p*-value for difference	0.02 *	0.2	0.13	0.85	0.36	0.18
Total (n = 29)	42,202.83	13,365.83	0.48	0.44	220,476.18	35,179.29

Note: All tests of means were conducted using t-tests assuming unequal means. All tests of medians were conducted using a non-parametric K-sample test on the equality of medians with the chi-squared test statistic computed with a continuity correction. * *p* < 0.05, ** *p* < 0.01, *** *p* < 0.001.
